# Evaluation of Multi-Target Genotyping (ITS-*hsp70*-*cpb*) for Detecting Population Heterogeneity Within Mediterranean *Leishmania infantum*, with a Focus on Zymodeme MON-24

**DOI:** 10.3390/pathogens15020145

**Published:** 2026-01-29

**Authors:** Trentina Di Muccio, Daniele Tonanzi, Gert Van der Auwera, Eleonora Fiorentino, Luigi Gradoni, Marina Gramiccia, Giuseppe La Rosa

**Affiliations:** 1 Unit of Vector-Borne Diseases, Department of Infectious Diseases, Istituto Superiore di Sanità, Viale Regina Elena 299, 00161 Rome, Italy; eleonora.fiorentino@iss.it (E.F.); luigi.gradoni@gmail.com (L.G.); gramiccia.marina@gmail.com (M.G.); 2 European Union Reference Laboratory for Parasites, Department of Infectious Diseases, Istituto Superiore di Sanità, Viale Regina Elena 299, 00161 Rome, Italy; daniele.tonanzi@iss.it (D.T.); giuseppe.larosa2025@outlook.it (G.L.R.); 3 Institute of Tropical Medicine, 2000 Antwerp, Belgium; gvdauwera@itg.be

**Keywords:** *Leishmania infantum*, heat shock protein 70, cysteine proteinase B, ITS, molecular diagnosis, sequencing, zymodeme MON-24, Mediterranean area

## Abstract

*L. infantum* and *L. donovani*, distinct species in the *L. donovani* complex, show high phenotypic and genotypic polymorphism and share molecular traits. Therefore, genotyping by a single molecular target can give uncertain results. This study focuses on genotyping a set of *L. donovani* complex strains, including 18 zymodemes classified according to Montpellier nomenclature (ZMONs) and different clinical forms, by internal transcribed spacer (ITS), heat shock protein 70 (*hsp70*), and cysteine proteinase b (*cpb*) sequencing to evaluate their ability in species discrimination. We found an unexpected *L. infantum hsp70* variability, with 8 sequence variants. *Cpb*-PCR could not distinguish *L. donovani* complex species, due to a *L. infantum* intraspecific allelic (*cpbEF*) polymorphism. By combining *ITS*-*hsp70-cpb* sequence variants, we obtained different genotypes. *ITS(A)-hsp70inf(2)-cpbE* identified 69.9% of *L. infantum* strains representing 12 ZMONs from Mediterranean visceral and cutaneous cases, *ITS(A)-hsp70inf(2)-cpbF* identified a non-ZMON-1 cluster. Four genotypes represented ZMON-24: *ITS(A, B)-hsp70(Y)-cpbF* identified a cutaneous cluster from Italy and North Africa, *ITS(A, Lombardi)-hsp70(2)-cpbE* identified cutaneous and visceral cases from Mediterranean areas. We believe this study contributes to an overview of *L. infantum* variant populations, and to the discussion on diagnostic targets, in single or multi -target-based approaches, to identify *Leishmania* populations in the Mediterranean area.

## 1. Introduction

Leishmaniasis is considered a neglected infectious disease that affects, kills or disables millions of people worldwide [1,2]. The disease affects both humans and animals and is caused by protozoan parasites of phagocytic cells belonging to *Leishmania* genus (Kinetoplastida, Trypanosomatidae) transmitted by bite of several sandfly *Phlebotomus* species. In the WHO European Region, the estimated number of visceral (VL) and cutaneous (CL) leishmaniasis cases is 1100–1900 and 10,000–17,000 cases per 100,000 population, respectively [3]. In this region, leishmaniasis is considered emergent and re-emergent due to different epidemiological factors such as human immunodepression, human migration, transport of infected animals, climate and environmental changes which affect the vectorial capacity and geographic distribution of sandfly populations [4,5].

*Leishmania infantum* (*L. donovani* complex) is the main agent of zoonotic VL in Mediterranean areas where dogs represent the main reservoir hosts for human infection. It has long been considered a viscerotropic species, but many cutaneous cases have been recorded throughout the Mediterranean basin which were mostly attributed to dermotropic variants of *L. infantum*, according to isoenzyme based-classification of parasite populations known as Montpellier zymodemes (ZMON) [6,7,8,9,10].

Clinical manifestations of the human leishmaniasis are highly polymorphic including asymptomatic infections, spontaneously healing localized skin nodules or cutaneous ulcers (cutaneous leishmaniasis, CL), multiple nodules (diffuse cutaneous leishmaniasis, DCL), infection of the mucous membranes (mucosal leishmaniasis, ML), and serious systemic visceral forms (visceral leishmaniasis, VL) which have a high mortality rate if not treated. Hosts’ immune response and cell-mediated immunity establish the outcome of the disease. Immunodeficiency syndromes from human immunodeficiency virus (HIV) infection, immunosuppressive treatments, and organ transplantation or malnutrition in childhood are major risk factors for VL by *L. infantum* [11,12,13]. Occasionally, VL may also exhibit epidemic characteristics among immunocompetent adults without natural acquired immunity to the parasite [3,8,14].

*Leishmania* identification has important implications both clinical and epidemiological in disease control and prevention, considering the emergence and re-emergence of *Leishmania* in different Mediterranean areas and the possible introduction of new species and variants [15,16,17,18,19,20,21,22]. Some genetic evidence can affect the molecular identification of *Leishmania* species. In fact, in this area, *L. infantum* shows a high polymorphism highlighted in the past by Multilocus Enzyme Electrophoresis (MLEE) typing [6,7,8,9,10] and more recently by molecular and genomic typing tools, such as Multi Locus Sequence Typing (MLST), Multilocus Microsatellite Typing (MLMT), and Next Generation Sequencing (NGS) [23,24,25,26,27,28,29,30,31]. Moreover, different hybrids such as *L. infantum/L. major* and *L. infantum/L. donovani* have been also demonstrated [32,33]. Therefore, the single marker-based approach can display uncertain results in close species, such as *L. infantum* and *L. donovani,* which are not clearly distinguished from a genetic point of view, given that they are known to form a *continuum* of populations in *L. donovani* species complex [34,35]. Consequently, the validation of different molecular targets and approaches should be considered as an ongoing process, and it should be evaluated in different epidemiological and geographical settings, considering phenotypic and genotypic polymorphisms, as well as the high genomic plasticity and adaptive capacity of *Leishmania* parasites [36,37].

For these reasons, the aim of this study was to genotype *L. donovani* complex strains by the most widely used diagnostic targets such as internal transcribed spacers ITS1 and ITS2 (ITS), heat-shock protein 70 (*hsp70*) and cathepsin L-like cysteine proteinase B (*cpb*) genes [38,39,40,41,42] also by combining *ITS-hsp70-cpb* sequence variants to gain more informative genetic data on *L. infantum* populations. For that, we selected a representative set of *L. donovani* complex strains, isolated over a 30-year period from CL and VL cases, occurred either as sporadic clinical episodes or during disease outbreaks, in order to account not only for the intraspecific variability, but also to rule out geographical bias due to the focal epidemiological aspect of this disease. We believe that our findings can contribute to the discussion on the variability and utility of these specific molecular targets, in single and multi-target-based approaches, in identifying *Leishmania* populations in the Mediterranean region.

## 2. Materials and Methods

### 2.1. Ethics Statement

Ethical review and approval were not required, as genome sequence analysis on *Leishmania* isolates is part of surveillance activities, conducted within the scope of the public health practice in Italy. Human clinical samples were collected and analyzed within the mandate of the Department of Infectious Diseases of Istituto Superiore di Sanità (ISS) as conferred by the Italian Ministry of Health (Protocol number 33122-14/10/2020-DGPRE-DGPRE-P ‘Prevention and control of Leishmaniasis in Italy’). Patient’s informed written consent was obtained from all subjects at the time of the clinical examination. Patient information was anonymized and de-identified prior to analysis, according to the European Union General Data Protection Regulation (Regulation (EU) 2016/679) (https://eur-lex.europa.eu/eli/reg/2016/679/oj, accessed on 30 November 2023) adopted by ISS (www.iss.it) and in agreement with the Data Protection Officer of ISS.

### 2.2. Samples

A total of 88 strains has been studied. Eighty-six strains were previously isolated, cultured and cryopreserved at Istituto Superiore di Sanità in Rome, during a period between 1982 and 2017. *Leishmania* strains originated from different geographical areas, from the main medical forms of Leishmaniases (VL, CL, and DCL), isolated from human cases of all clinical categories (infants, adults, immunocompetent and immune compromised patients); a few strains from animal hosts (dog, cat and *Phlebotomus* sand flies) were included. As shown in Table S1A, all the strains have been classified by ITS sequencing according to Kuhls et al., 2005 and Chicharro et al., 2013 [14,43]: 82 *L. infantum* strains from Mediterranean areas (*n* = 68 from Italy, *n* = 10 from Spain, *n* = 1 from Malta, *n* = 3 from North Africa) and 4 *L. donovani* strains from Africa (Table S1A). Furthermore, 2 reference strains were included in the analysis: *L. infantum* MHOM/DZ/82/LIPA59-ISS182 from Algeria, and *L. donovani* MHOM/IN/80/DD8-ISS46 from India. A total of 72 out 88 strains, previously typed by MLEE, were representative of 15 *L. infantum* ZMONs and 3 *L. donovani* ZMONs [7,8]. In Table S1B, we summarized the analyses (MLEE, sequencing, RFLP, dendrogram, PCoA) performed for each strain.

### 2.3. DNA Extraction and Amplification

The genomic DNA from 10^6^ promastigote cultures in EMTM [44] was prepared by the DNA extraction protocol of the Maxwell^®^ 16 Cell DNA Purification Kit (Promega, Medison, WI, USA) following the manufacturer’s instructions. The DNA concentration was measured spectrophotometrically. The DNA was stored at −80 °C until use.

All the PCRs were performed in volumes of 50 µL of PCR mixture (GoTaq Green Master Mix, 2X Promega) using 2 µL of DNA (5–10 ng) and 1 µL of the appropriate primer pair for each of the three markers selected. The PCRs were performed in the C1000 Thermal Cycler (BIORAD, Hercules, CA, USA). A 2% agarose gel was used to verify the amplified product size.

The ITS1-5.8S-ITS2 cluster (here named ITS) was amplified by LITSV and LITSR primers, according to the amplification conditions described by El Tai et al., (2000) [45] with some modifications: initial denaturation at 95 °C for 5 min followed by 34 cycles consisting of denaturation at 95 °C for 20 s, annealing at 53 °C for 30 s, and extension at 72 °C for 1 min followed by a final extension cycle at 72 °C for 10 min in a single PCR reaction.

For all strains, the F-fragment of the *hsp70* gene was amplified by using 20 pmoles of each primer F25 and R1310 [40]. The *hsp70*-F fragment RFLP assay was performed to discriminate *L. donovani* and *L. infantum*, as proposed by Montalvo et al. (2012) [40]. The digestion was performed in a total of 20 μL containing 5 μL of the PCR products in 1x buffer provided by the manufacturers, and 5U restriction enzyme *Mlu*I (Promega). The reactions were incubated at 37 °C overnight and analyzed by electrophoresis in 3% agarose gel (Promega). When uncertain, the PCR-RFLP assay was repeated to ensure the repeatability of the results. 

Different copies of the *cpb* gene were amplified by using specific primers CpbEF-for and CpbEF-rev. This PCR was described as discriminative between the *L. donovani* and *L. infantum* species, amplifying the copies *cpbF* (741 bp) and *cpbE* (702 bp), respectively [46].

### 2.4. Sequencing and Genetic Analysis

The three markers (ITS, *hsp70* and *cpb*) were amplified in triplicate from each strain and the independent PCR products were sequenced by using the same primers used in the amplification reactions in both directions. The final forward/reverse consensus was produced by CLC Main Workbench software package ver.23 (Qiagen Aarhus A/S). Only the sequences showing reproducible results were submitted in GenBank at NCBI. 

The ambiguous nucleotides have been codified using IUPAC code. The heterozy-gous positions, identified by a double peak in the chromatogram, were distinguished from a poor sequence quality if the peaks were of similar height, or the lower peak was at least 50% of the highest or considerably higher than the background noise in the rest of the chromatogram, and if the double peak was present in sequencing results from both directions. All the *hsp70* and *cpb* sequences were aligned using the multiple alignment tool implemented in CLC package and were manually adjusted to maximize identity in presence of microsatellites (ITS) and indels (*cpb*). Before analysis, the multiple alignments were trimmed at the 3’ and 5’ ends to the sequence fragment shared by all samples.

Each ITS sequence was typed by comparing it to an “ITS-types” set of *L. infantum* and *L. donovani*, previously classified by using the sequence polymorphisms of the 12 microsatellite regions, including four sites in ITS1 and eight sites in ITS2 [14,43]. The *hsp70* and *cpb* sequences were compared to well-characterized reference sequences [29,47,48] retrieved from GenBank at NCBI.

A dendrogram of *cpb* was built to evaluate the genetic relationships among the *L. donovani* complex strains with respect to this molecular target. Sequences of *cpbEF* were retrieved from Genbank with BlastN, using as query the sequences obtained in this study. Sequences that showed a similarity of less than 95% with the query, containing frameshift mutations or several ambiguous nucleotides, were not selected for the analysis. Multiple alignment including 61 sequences from Genbank and the 23 from this study were used. The dendrogram was based on p-distances by using the Neighbor-joining method with MEGA version 7.0 (megasoftware.org). The bootstrap value for each node was computed using 2000 replicates.

The Principal Coordinates Analysis (PCoA) was performed to investigate the rela-tionships among the *Leishmania* strains sub-set including the 23 strains for which ITS, *hsp70* and *cpb* sequences were available. The PCoA does not need any a priori assumption on substitution model for nucleotide evolution and each nucleotide position is treated as a phenetic character with two characters states (presence “1” and absence “0”). The PCoA was performed by Past software version 4.14 [49] using the Jaccard’s index to compute the phenetic similarity matrix among strains. The default value of Transformation Exponent (c = 2) was applied. The input binary data table used to compute the PCoA was produced including as character each variable nucleotide position present in the multiple ITS, *hsp70*, and *cpb* alignment and codifying as presence/absence each alternative nucleotide (Table S3). The Indel in *cpb*, spanning up to 39 bp, was codified as a single character (i.e., single mutational event) and the same was for variable microsatellites in ITS (i.e., occurrence of alternative repeat was considered as a single event). The multidimensional space produced by the analysis and describing the relationships among the *Leishmania* strain was summarized by the two graphics defined by the three main coordinates (i.e., axes). The sequences of *L. infantum* MHOM/FR/78/LEM75 strain available in GenBank (accession numbers: AJ634339—ITS, LN907838—*hsp70* and AY896781—*cpbE*) were also included in the analysis as reference.

## 3. Results

The genetic variability shown by the ITS, *hsp70* and *cpb* sequences was used to address key diagnostic, epidemiological and population genetic issues regarding our 88 strains. The GenBank accession numbers are listed in Table S1A.

ITS displayed well-known *L. infantum* ITS-A, ITS-B and ITS-Lombardi genotypes, widespread in Mediterranean areas including North Africa, and on the other hand *L. donovani* ITS-E and ITS-H from East Africa and India [14,43]. Four ITS-B var, A/B var, E var sequence variants were observed (Table S1A).

Eighty-eight *hsp70* sequences were obtained. The consensus sequences were identified as belonging to *L. donovani* complex species by a Blast search in GenBank at NCBI. The multiple sequence alignment of 1019 bp displayed a genetic variability due to nine polymorphic sites that gave 9 sequence variants with respect to the sequence of the reference strains *L. donovani* MHOM/KE/55/LRC-L53 (MN728785) and *L. infantum* MHOM/FR/78/LEM75 (LN907838) (Tables S1 and S2). The percentage of identity in intraspecific pairwise comparison was 100% in *L. donovani* and spanning from 99.75% to 100% in *L. infantum*. The sequence variants *hsp70don(1)* (*n* = 5/5, 100% of the *L. donovani* strains) and *hsp70inf(2)* (*n* = 67/83, 80.7% of the *L. infantum* strains) displayed 100% identity with *L. donovani* and *L. infantum* reference sequences, respectively. In contrast, the sequences *inf(3–9)* displayed different variant nucleotide positions (Table S2).

The most relevant sequence variants were *inf(6–9),* which identified 48% (*n* = 12/25) of the *L. infantum* ZMON-24 strains. They displayed variant nucleotides in 3 positions (266, 370, and 888) and were characterized by a heterozygous condition with Y (T/C) in the species-specific site 370, present in one of two *Mlu*I restriction sites (ACGCGT), proposed by Montalvo et al., (2012) [40] to discriminate *L. infantum* from *L. donovani* by RFLP assay (Table S2). Therefore, the reliability of this assay was evaluated on all the samples. The triple band pattern (117, 389, 780 bp), typical of *L. infantum* sp., was observed in 85.5% (*n* = 71/83) of the *L. infantum* strains. A double band pattern (117 and 1169 bp), typical of *L. donovani* sp., was observed in 100% (*n* = 5/5) of the *L. donovani* strains, as expected. In contrast, a *L. infantum/L. donovani* mixed pattern, with 4 restriction products (117, 389, 780 and 1169 bp) common to the two species, was found in 14.5% (*n* = 12/83) of *L. infantum* strains, referring to the sequence variants *inf(6–9)* (Figure S1). This pattern was due to the possible overlap of two *hsp70* alleles, in the heterozygous position Y (Figure S2). For this reason, we referred to the sequence variants *inf(6–9)* as *inf(Y)*.

The *cpb*-PCR of the 88 samples evidenced the presence of the copy *cpbE* (702 bp) in 67 out of 83 (80.7%) *L. infantum* strains; the copy *cpbF* (741 bp) was detected, not only in 5 *L. donovani* strains, as expected, but also in 16 *L. infantum* strains (19.3%), classified as zy-modems MON-24 (*n* = 12 strains), MON-187 (*n* = 1), MON-189 (*n* = 1), and MON-190 (*n* = 2) (Table S1A).

By combining the ITS-*hsp70* sequence variants and the copies *cpbE* and *cpbF*, 14 *ITS-hsp70-cpb* sequence variants were generated and here indicated as a “genotype” (Table 1). 

Geographical distribution of the genotypes A–H was depicted on the MicroReact Map. The whole dataset can be downloaded and explored interactively in the MicroReact platform (https://microreact.org/project/iss, accessed on 30 November 2025) [50]. The genotype C was found in 69.9% (*n* = 58/83) of *L. infantum* strains belonging to 12 zymodemes from Mediterranean areas. Furthermore, the genotype H was found in ZMON-187, ZMON-189, and ZMON-190 zymodemes. Interestingly, at least 4 genotypes (C, E, F, and G) represented ZMON-24: F and G identified CL cases from Italy and North Africa, instead the genotypes C and E identified VL and CL cases from Mediterranean areas. 

Consequently, in order to provide an accurate overview of the genetic relationships among the ZMON-24 strains, we sequenced *cpbE* and *cpbF* for 19 of them, along with one *L. infantum* ZMON-1 (ISS3176), ZMON-187, *L. donovani* ZMON-30 (ISS2440), and ZMON-18 (ISS50) strains (the sequence accession number in Table S1A). The alignment of 704 bp displayed a genetic variability due to sixteen polymorphic sites and one 39 bp gap that returned 7 sequence variants: *cpbF(1–4)* and *cpbE(1–3)*, 5 of them detected in ZMON-24 strains (column CpbEF Seq(var) in Table S1A). Most of the variability was due to the presence of *L. donovani* strains (ISS50 and ISS2440), differing in identity percentage from all the other *L. infantum* strains from 98.15% to 98.43%. The identity percentage in the intraspecific pairwise comparison was 100% in *L. donovani*, while it was between 99.29% and 100% in *L. infantum*. 

As shown in the dendrogram in Figure S3, our strains were grouped into seven clusters of identical sequences. The *L. infantum cpbE(1–3)* sequence variants clustered with the CL and VL *L. infantum* cluster (ZMON-1, ZMON-24, ZMON-80) from the Mediterranean area (France, Spain, Tunisia, and Algeria), previously described [47,48]. On the contrary, the sequences *cpbF(1)* clustered with *L. donovani* strains from East Africa, whereas the sequence variants *cpbF(2–4)* were grouped with *L. infantum* strains from North Africa, among which ZMON-24 CL strains (see Table S5).

**Figure 1:**
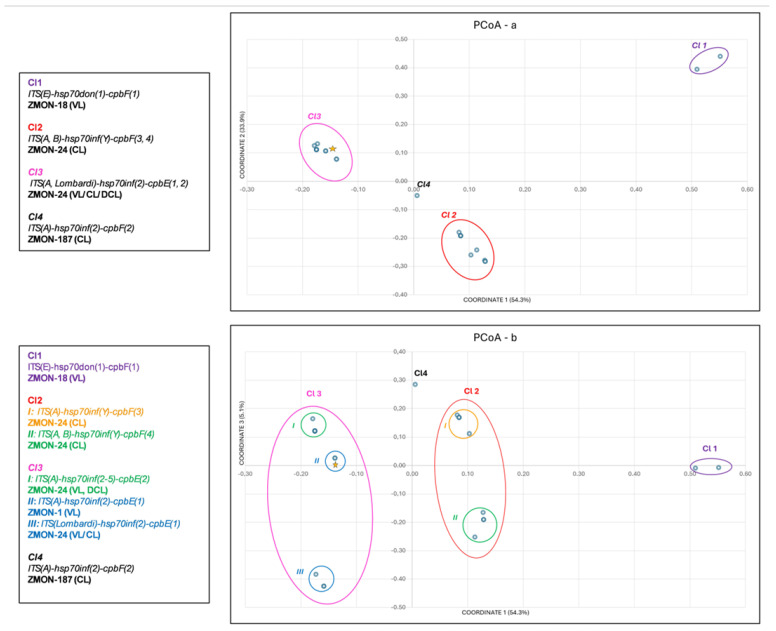
(**a**,**b**). Multidimensional analysis (PCoA) displaying the most probable groupings of 24 *L. donovani* complex strains, including the reference strain *L. infantum* MHOM/FR/78/LEM75 (star). (**a**) The coordinates 1 vs. 2 (total variance 88.2%) show 4 clusters (Cl 1–4), depicted by ellipses, including the main *ITS-hsp70-cpb* sequence variants; (**b**) the coordinates 1 vs. 3 (total variance 39.0%) show 5 sub-clusters Cl2 (I–II) and Cl3 (I–III), depicted by circles; the color of the circles indicates the geographic origin: green, Italy and North Africa; blue, Europe; orange, Italy; purple, East Africa. All the codes ISS of our 23 strains have been shown in Table S4.

By looking at multidimensional PCoA, we observed the genetic relationships among these 23 sequence variants of *ITS-hsp70-cpb* by studying three coordinates that accounted for 93.3% of the total variance (coordinate 1 = 54.3%, coordinate 2 = 33.9% and coordinate 3 = 5.1%) (Table S3). The most probable groupings are shown in Figure 1a,b. The analysis obtained by using the coordinates 1 vs. 2 (total variance 88.2%) (Figure 1a) grouped the strains into 3 main clusters: Cl 1 including *L. donovani* strains, characterized by *ITS(E)-hsp70don(1)-cpbF(1)*; Cl 2, including only cutaneous *L. infantum* ZMON-24 strains from Italy and North Africa, characterized by *hsp70inf(Y)* and *cpbF(3–4)*; Cl 3, in-cluding ZMON-24 VL, CL and DCL, and ZMON-1 VL cases (including MHOM/FR/78/LEM75 *L. infantum* reference strain, ZMON-1), characterized by *hsp70inf(2)* and *cpbE(1–2)*. Finally, the ZMON-187 strain (Cl 4) was separated from the other ones, due to the unique combination of *hsp70inf(2)* with *cpbF(2).*

Furthermore, in the graphic obtained by the coordinates 1 vs. 3 (total variance 39.0%) (Figure 1b), Cl 2 was divided in 2 sub-clusters: I, represented by CL strains from Italy, exhibiting *cpbF(3)*, II represented by strains from Italy and North Africa, exhibiting *cpbF(4)*. Cl 3 resulted divided into 3 sub-clusters: I and II, including European *L. infantum* ZMON-1 and ZMON-24 from Italy and Spain, respectively, all strains with the *cpbE(1)*, while the cluster III included all the *L. infantum* ZMON-24 from Italy and North Africa, with *cpbE(2).* Again, the Italian *L. infantum* ZMON-187 strain remained separated. 

## 4. Discussion

Our study focused on integrating genetic variability data by sequencing diagnostic targets that have been extensively studied in *Leishmania*, such as ITS, *hsp70*, and *cpb*, to identify strains from Mediterranean areas. Moreover, we described different *Leishmania* populations with a multi-targets-based approach by combining the sequence variants of *ITS-hsp70-cpb* and obtaining 8 “genotypes” (A–H) (Table 1). 

By examining each marker individually, we can highlight some aspects. Our sequence analysis of ITS target allowed a *Leishmania* species classification of our strains, showing well known geographic *L. infantum* populations (genotypes ITS-A, ITS-B, and ITS-Lombardi), widespread in the Mediterranean area including North Africa, and *L. donovani* populations (genotypes ITS-E and ITS-H) from East Africa and India [14,43]. There was no evidence of a clear correlation between ITS genotypes and either enzyme populations, hosts, or clinic.

The *hsp70* gene sequence is widely accepted as a tool for discrimination of medically important *Leishmania* species from the Old and New World, due to the high conservation observed within *L. donovani* complex genomes from different geographic regions [40,41,51]. In our study, we found an unexpected intraspecific variability in the Mediterranean *L. infantum* strains, proven by 8 *hsp70* F-fragment sequence variants, while confirming *hsp70* its validity as a genotyping target of *Leishmania* species genotyping target. The most represented variant *hsp70inf(2)*, displaying 100% identity with the *L. infantum* LEM75 reference strain, identified 80.7% of *L. infantum* strains, including all the 15 zymodemes studied. However, we detected 4 sequences (i.e., *inf(6–9)*), referred to as *inf(Y)*, that are characterized by their common heterozygous position (Y = C/T) at the species-specific site distinguishing *L. donovani* from *L. infantum*. Standard conditions have been applied to perform reliable sequences and exclude sequencing artefacts (see Section 2.4); moreover, the RFPL results supported the proof that this position could highlight the presence of two *hsp70* alleles, as already reported in several *Leishmania* species [32,52]. However, heterozygosity should be further investigated. Therefore, these strains will be analyzed by a low-coverage whole-genome sequencing (WGS) to evaluate the possible hybrid status. 

A BLAST search in GenBank at NCBI (https://blast.ncbi.nlm.nih.gov/Blast.cgi, accessed on 28 July 2023) showed the originality of this *hsp70*-F fragment sequence variants that, according to our analysis, was specific of a ZMON-24 sub-cluster of Italian and African strains (*n* = 12) with cutaneous feature. Therefore, the sequence *hsp70inf(Y)* and/or the RFLP assay, which showed a reproducible and specific pattern, can represent possible rapid typing assays able to identify a *L. infantum* ZMON-24 sub-cluster. The correlation between this *hsp70inf(Y)* sequence variant, the cutaneous presentation, and geographic origin should be evaluated with additional samples. Meanwhile, we found, from previous works by Gritti et al., (2023, 2025) [53,54] in the Emilia-Romagna region (Italy), some original *L. infantum hsp70* fragment sequence variants from CL cases, displaying heterozygous position Y, consistent with ZMON-24, historically present in this area [7,8].

The *cpb* gene is considered an efficient marker to discriminate between *Leishmania* species [38,54], based on PCR-size polymorphism. However, we highlighted intraspecific polymorphism (*cpbE* and *cpbF*) within our *L. infantum* strains. In fact, in some *L. infantum* zymodemes (i.e., ZMON-24, ZMON-187, ZMON-189, ZMON-190) we detected (by *cpb*-PCR assay) the copy *cpbF*, known to be specific of *L. donovani*, as also described in some *L. infantum* populations in Tunisia, Algeria, and recently in North Italy [48,54,55,56,57]. Therefore, the *cpb*-PCR size polymorphism is not a useful assay to distinguish *L. donovani* from *L. infantum*. The sequencing of 23 *L. infantum* and *L. donovani* strains displayed 7 *cpbEF* sequence variants (Table S1A). As we showed in the *cpb* dendrogram (Figure S3), the sequences variants *cpbE(1–3)* indicated an *L. infantum* cluster of strains from Mediterranean areas, whereas *cpbF* was not exclusive of *L. donovani* sp., but indicated a *L. donovani* complex cluster, with the sequence variants *cpbF(1)* and *cpbF(2–4)* identifying *L. donovani* and *L. infantum* strains, respectively (Table S5). Therefore, the validity of *cpb* sequencing as a *L. infantum* population genetic marker should be evaluated by a more comprehensive study.

The multi-target-based diagnostic approach, obtained by combining the *ITS-hsp70* sequence variants and the copies *cpbE* and *cpbF*, allowed a more accurate identification of *Leishmania* populations than the single-target-based approach. We found that the geno-type C was the most represented in our samples, identifying 68.2% of *L. infantum* strains, which belonged to 12 zymodemes from the Mediterranean area (see Microreact Map at https://microreact.org/project/iss) (accessed on 30 November 2025) [50]. On the contrary, *cpbF*, in combination with the sequence variant *hsp70inf(Y)* (genotypes F and G), detected a ZMON-24 sub-cluster of CL cases, not ITS(Lombardi). In addition, *cpbF*, in combination with the reference sequence *hsp70inf(2)* (genotype H), identified a non-ZMON-1 cluster (i.e., ZMON-187, ZMON-189, ZMON-190). Due to the rarity of these latter zymodemes [7,8,9,10], it is not easy to increase the number of samples for further analyses in an effort to correlate this genotype to specific zymodemes or clusters of zymodemes. However, as part of collaborations with the Leishmaniasis centers of the Mediterranean Countries, we hope to further explore this issue. 

With respect to the correlation of ZMONs with genotypes *ITS-hsp70-cpb*, we ob-served that *L. infantum* ZMON-1 was not very heterogeneous, being represented by gen-otype C, detected in 89.3% (*n* = 25/28) of strains (identical to the reference strain LEM75). In contrast, ZMON-24 (*n* = 25 strains) seems to be a polymorphic zymodeme, being identified by 6 genotypes, of which the most represented were C, E, F, and G (Table 1).

Moreover, the multi-target approach unveiled additional genetic populations in ZMON-24, whose relationships, also with respect to ZMON-1 and *L. donovani*, were inferred by PCoA analysis. Heterogeneous clinical, geographic and genetic aspects of the ZMON-24 sub-populations were evaluated: *ITS(A)-hsp70inf(2)-cpbE(1, 2)* identified all the most serious clinical cases, such as VL (with concomitant CL in HIV co-infection) and DCL, along with *ITS(Lombardi)-hsp70inf(2)-cpbE(1)*, including both CL and VL cases, from all over the Mediterranean area. These genotypes were closer, if not identical, to those of ZMON-1. In contrast, *ITS(A, B)-hsp70inf(Y)-cpbF(3, 4)* included only ZMON-24 CL cases. The *cpbF* sequence variants further differentiated the exclusively Italian *L. infantum* strains, *cpbF(3)*, from those in cluster Italy and North, *cpbF(4)* (Figure 1). A low-coverage whole-genome sequencing (WGS) of our strains, in particular of ZMON-24, is currently being performed to characterize these *L. infantum* populations and to evaluate its consistency with our typing by *ITS-hsp70-cpb* in a more comprehensive study. 

Regarding the clinical correlation of ZMON-24 findings, different zymodemes are related to changes in biological features, such as clinical manifestation and virulence [58,59]. ZMON-24, widely spread in South Europe and North Africa, has been considered for a long time an isoenzymatic population with a dermotropic character, causing VL only in HIV-co-infected patients, and sporadically in children [59,60,61,62,63]. Researchers in previous studies, showed that some VL and CL ZMON-24 strains from Tunisia and Algeria, ana-lyzed by MLMT and *cpb* sequencing [47,48,64], could be divided into two genetic pop-ulations with clinical correlation. They also discussed whether this was a general trait of these populations, or a spurious association caused by geographical sampling bias. Our study, by different molecular targets, seems to confirm these observations. We also draw some conclusions from a speculative point of view. 

First, in our sample set, *L. infantum* ZMON-24 includes two main genetic populations correlated to clinical characteristics. *Hsp70inf(Y)-cpbF* population could be a distinct evolutionary lineage within the *L. donovani* complex, originated in Africa and spread across the Mediterranean region (e.g., Algeria, Tunisia, Morocco, and Italy). This population could be a dermotropic variant of a normally viscerotropic species, although it inevitably causes VL in patients with HIV/*Leishmania* co-infections, such as ISS417 strain [7,57,60,64]. In contrast, the ZMON-24 population *hsp70inf(2)-cpbE*, closer to ZMON-1, appears virulent, causing VL in adult and children, immunocompetent and, more frequently, HIV-positive patients (8 strains) (Table S1A). These conclusions lead to the hypothesis that the clinical manifestation of ZMON-24 could also depend on its genetics, in addition to the host’s immunological state. Additional strains of different ZMON-24 CL and VL genotypes need to be investigated in order to better understand the role of parasite fitness and host susceptibility. 

Second, it must be considered that discriminatory power of MLEE is limited, because genotypes are assayed indirectly, since synonymous nucleotide substitutions may not be observed and different allozymes may have coincident mobility. In contrast, despite identical genotypes, post-translational modifications may influence the electrophoretic mobility of the proteins [65]. Consequently, the ZMON-24 strains with genotype *ITS(A, Lombardi)-hsp70inf(2)-cpbE(1)* could be considered closer to *L. infantum* ZMON-1 viscerotropic strains, as highlighted by sequence analysis. 

This study has some limitations. As already mentioned, it should be noted that our interpretation of the clinical–genetic correlations of the different molecular *Leishmania* populations was somehow speculative. Indeed, the correlation of specific molecular traits with zymodemes or clinical features requires more robust evidence, in terms of number of strains, and molecular approaches. More robust approaches will also be needed to assess the heterozygous status of some ZMON-24 strains. Moreover, due to the possible geographical sampling bias of our clinical samples, which are a retrospective collection (not representative of all the Mediterranean endemic areas), the actual spread of the described genotypes is not comprehensive. Nevertheless, this study can provide an overview of different genotypes, which were obtained by a multiple-target-based approach, and address the significance of some *L. infantum* sequence variants for each target detected in Mediterranean areas, linking them with specific zymodemes. 

## 5. Conclusions

The wide heterogeneity of the Mediterranean *L. infantum* strains returned a molecular polymorphism through well validated species-specific molecular markers. Our results have confirmed the ITS and *hsp70* validity as markers in *Leishmania* species genotyping; however, new *L. infantum hsp70* variants have been described, and their correlation with specific *L. infantum* populations should be further investigated. On the contrary, PCR-*cpb* size polymorphism was found to be an ineffective species-specific marker in the Mediterranean area for the *L. donovani* complex species, showing an *L. infantum* intra-specific size polymorphism due to the detection of both copies *cpbE* and *cpbF*. However, a more comprehensive study will test whether the *cpb* sequencing can be used as a geographic marker for *L. infantum*. Although our multi-target approach does not allow for in-depth identification compared to other molecular approaches, it can easily and quickly distinguish several *Leishmania* populations. Finally, due to the *Leishmania* genetic heterogeneity detected by *hsp70* and *cpb*, the leishmaniasis typing protocols should, in our view, provide a combination of these markers, associated with ITS, in the Mediterranean areas.

## Figures and Tables

**Table 1:** Genotypes obtained by combining ITS and *hsp70* sequence variants and the *cpb* copies, *cpbE* and *cpbF*, of 88 Mediterranean *Leishmania* strains.

Sequence Variant *ITS/hsp70/cpb*	Genotype	ZMON	Clinic	Origin	N. Strains
*L. donovani*					
***ITS(H)-hsp70don(1)-cpbF***	A	2	VL	India	1
***ITS(Evar)-hsp70don(1)-cpbF***	unique	18	VL	Africa	1
***ITS(E)-hsp70don(1)-cpbF***	B	30	VL	Africa	3
***L. infantum***					
***ITS(A)-hsp70inf(2)-cpbE***	C	1, 24, 29, 34, 72, 80, 136, 185, 188, 201, 228	VL	Mediterranean area	58
***ITS(A)-hsp70inf(4)-cpbE***	D	1	VL	Italy	2
***ITS(Lombardi)-hsp70inf(2)-cpbE***	E	24	VL, CL	Spain	3
***ITS(A)-hsp70inf(Y)-cpbF***	F	24	CL	Italy	8
***ITS(B)-hsp70inf(Y)-cpbF***	G	24	CL	Italy, Africa	3
***ITS(A/B var)-hsp70inf(Y)-cpbF***	unique	24	CL	Africa	1
***ITS(A)-hsp70inf(5)-cpbE***	unique	24	DCL	Italy	1
***ITS(A)-hsp70inf(2)-cpbF***	H	187, 189, 190	VL, CL, DCL	Italy, Spain	4
***ITS(A/B var)-hsp70inf(2)-cpbE***	unique	78	VL	Italy	1
***ITS(Bvar)-hsp70inf(2)-cpbE***	unique	1	VL	Italy/Africa	1
***ITS(A)-hsp70inf(3)-cpbE***	unique	1	VL	Italy	1
Total					88

In red, the most represented *L. infantum* genotypes. The genotypes A–H referred to at least two strains or did not present any sequence variant with respect to the reference sequence (i.e., genotype A). In any other case, we considered them as a “unique” genotype.

## Data Availability

The sequences reported in this paper were deposited in GenBank database: Accession Numbers shown in Table S1.

## Supplementary Materials

The following supporting information can be downloaded at: https://www.mdpi.com/article/10.3390/pathogens15020145/s1, Table S1: (A) Eighty-eight *L. donovani* complex strains analyzed in this study with information about geographic origin, clinic, biochemical and molecular characterization, and accession numbers at GenBank, (B) Overview of the analyses (MLEE, se-quencing, RFLP, dendrogram, PCoA) performed for each strain; Table S2: Description of the polymorphic nucleotide positions of the *hsp70* F-fragment sequence of 88 *L. donovani* complex strains from the Mediterranean area; Table S3: Data used to analyze the relationships among 24 *Leishmania* donovani complex strains, (A) Multiple Alignment, (B) Binary Table, (C) Jaccard’s in-dex, (D) PCoA coordinates; Table S4: Addendum of the Figure 1 legend; Table S5: Sequence-related information of the *Leishmania* strains present in the *cpb* dendrogram retrieved from Genbank; Figure S1: *Hsp70* F-fragment PCR-RFLP analysis; Figure S2: *Hsp70* sequence electropherograms with indication of *Mlu*I restriction site and heterozygous site; Figure S3: Dendrogram of *cpb* sequences with indication of sequence types E1–E2 and F1–F4. https://microreact.org/project/iss (accessed on 30 November 2025).
