# Supplementary material for: Evaluation of Multi-Target Genotyping (ITS-hsp70-cpb) for Detecting Population Heterogeneity Within Mediterranean Leishmania infantum, with a Focus on Zymodeme MON-24
Source: Pathogens. 2026 Jan 29;15(2):145. doi: 10.3390/pathogens15020145 (PMC12942746; doi:10.3390/pathogens15020145)
Supplement: Supplementary file 1 [file pathogens-15-00145-s001.zip › Table_S2.pdf]

Table S2. Description of the polymorphic nucleotide positions of the hsp70 F-fragment sequence of 88 *L. donovani* complex strains from the Mediterranean area.

Reference *Leishmania* sp. WHO code	hsp70 Seq (var)	Variant nucleotides positions									ZMON	N. strains
		266	370*	423	474	854	888	892	978	1082		
L. donovani **MHOM/KE/55/LRC**		G	C	G	G	C	G	C	C	A		
	don(1)	.	.	.	.	.	.	.	.	.	2, 18, 30	5
L. infantum **MHOM/FR/78/LEM75**		A	T	G	G	C	G	C	C	A		
	inf(2)	.	.	.	.	.	.	.	.	.	all§	67
	inf(3)		.	K	R	.	.	.	.	.	1	1
	inf(4)	.	.	.	.	Y	.	Y	S	.	1	2
	inf(5)	.	.	.	.	.	.	.	.	R	24	1
	inf(6)	N	Y	.	.	.	C	.	.	.	24	1
	inf(7)	R	Y	.	.	.	S	.	.	.	24	9
	inf(8)	R	Y	.	.	.	R	.	.	.	24	1
	inf(9)	N	Y	.	.	.	R	.	.	.	24	1
**Total**												88

Hsp70 sequence variants (Seq var) were found by the alignment of 88 *L. donovani* complex strains with the *L. donovani* MHOM/KE/55/LRC (MN728785) (*don1*) and *L. infantum* MHOM/FR/78/LEM75 (LN907838) (*inf2*) reference strains available in GenBank. Highlighted in red, the most represented polymorphic positions 266, 370, 888 in the variants *inf(6-9)* with respect to reference strains. * Species-specific position discriminating *L. donovani* and *L. infantum* according to [40]; § all the *L. infantum* zymodemes analyzed in this study.
