# Supplementary figures and images for: Evaluation of Multi-Target Genotyping (ITS-hsp70-cpb) for Detecting Population Heterogeneity Within Mediterranean Leishmania infantum, with a Focus on Zymodeme MON-24
Source: Pathogens. 2026 Jan 29;15(2):145. doi: 10.3390/pathogens15020145 (PMC12942746; doi:10.3390/pathogens15020145)

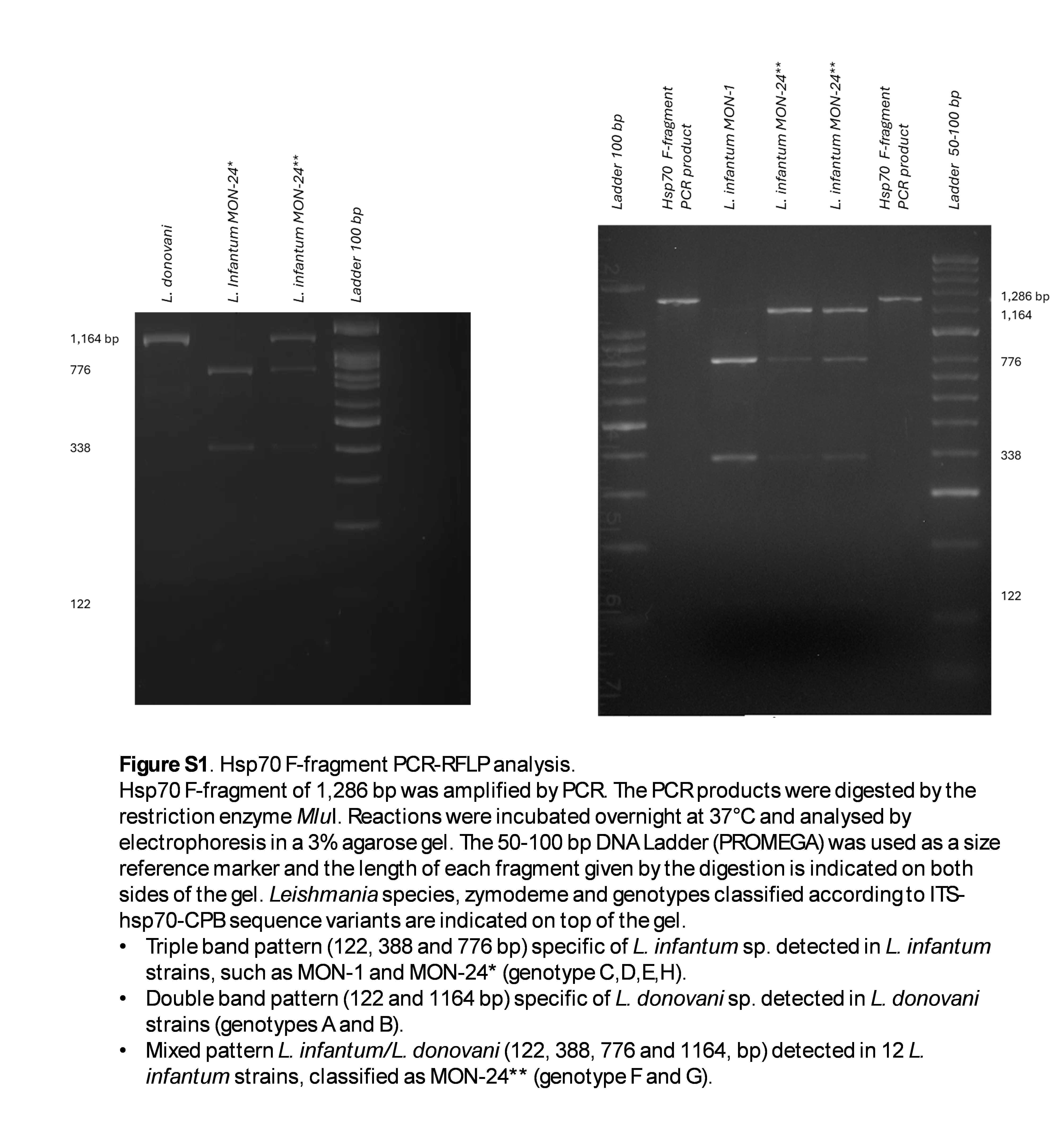

Supplement: Supplementary file 1 [file pathogens-15-00145-s001.zip › Figure S1.tif]

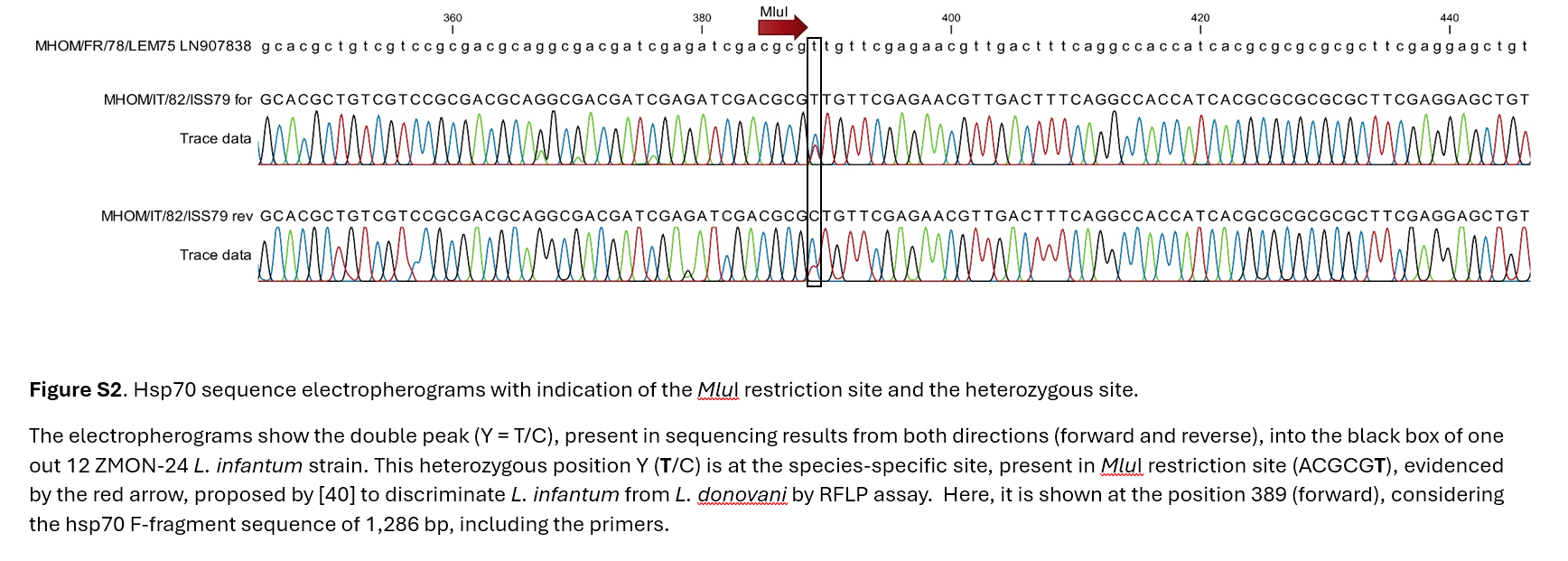

Supplement: Supplementary file 1 [file pathogens-15-00145-s001.zip › Figure S2.tif]
